# Early Attachment Disruption, Inflammation, and Vulnerability for Depression in Rodent and Primate Models

**DOI:** 10.3389/fnbeh.2018.00314

**Published:** 2019-01-07

**Authors:** Michael B. Hennessy, Patricia A. Schiml, Katelyn Berberich, Nicole L. Beasley, Terrence Deak

**Affiliations:** ^1^Department of Psychology, Wright State University, Dayton, OH, United States; ^2^Behavioral Neuroscience Program, Department of Psychology, Binghamton University, Binghamton, NY, United States

**Keywords:** early-life stress, maternal separation, attachment, depression, stress-induced sickness, inflammation, neuroimmune, animal models

## Abstract

Early experiments in nonhuman primates established the relation between disruption of filial attachment and depressive-like outcomes. Subsequent studies in rats and mice have been instrumental in linking depressive-like outcomes to disturbances in maternal behavior. Another aspect of attachment disruption, absence of the attachment object *per se*, may be studied more effectively in a different laboratory rodent—the guinea pig. Here, we discuss the rationale for using guinea pigs for this work. We then review guinea pig studies providing evidence for inflammatory mechanisms mediating both depressive-like behavior during separation as well as sensitization of stress responsiveness such as is thought to lead to increased vulnerability to depression at later ages. Finally, we discuss recent complementary work in adult monkeys that suggests cross-species generalizability of broad principles derived from the guinea pig experiments. Overall, the findings provide experimental support for human research implicating inflammatory mechanisms in the development of increased stress responsiveness and vulnerability to depression following attachment disruption and other forms of early-life stress. Specifically, the findings suggest inflammatory mechanisms may set in motion a cascade of underlying processes that mediate later increased stress responsiveness and, therefore, depression susceptibility.

## Early Studies of Early Experience

A recent PubMed search for the term “early life stress” yielded over 17,000 hits. The roots of this enormous body of scientific study can largely be traced back to three lines of research from the mid 20th century: studies of infantile “handling” in rats and mice, experiments in which infant monkeys were separated from their mothers, and observations of institutionalized children. The seminal rodent finding was the observation that brief, daily handling of preweaning rats improved measures of avoidance learning in adulthood (Levine et al., 1956). Additional studies by Levine, Denenberg, Ader and a number of other investigators soon documented that early handling produced various, often apparently adaptive, behavioral outcomes under stressful conditions, and reduced hypothalamic-pituitary-adrenal (HPA) responsiveness as well (e.g., Levine, 1956; Schaefer et al., 1962; Denenberg, 1964; DeNelsky and Denenberg, 1967; Ader, 1968). It gradually became apparent that many of the effects of handling were mediated by the mother’s treatment (e.g., licking) of the pups following their return to the nest (Barnett and Burn, 1967; Schreiber et al., 1977; Hennessy et al., 1982; Liu et al., 1997). Subsequent detailed analysis revealed neural and epigenetic mechanisms were a primary means through which maternal behavior exerts lasting consequences (Meaney et al., 1996; Cameron et al., 2008).

As the early handling studies began to appear in print, publications by Harlow, his students, and others showed that prolonged maternal separation or deprivation had devastating immediate as well as long-term consequences on emotional responsiveness and social behavior of nonhuman primates (Kaufman and Rosenblum, 1967; Mitchell, 1968; Harlow et al., 1971). Although extreme by today’s standards of animal welfare and acceptable experimental manipulation, these studies offered crucial empirical support for the work of other investigators examining children lacking normal maternal or substitute affectionate care in orphanages, hospitals, and other institutions (Spitz and Wolf, 1946; Robertson and Bowlby, 1952). The conclusion that such children were suffering serious emotional and depressive effects due to separation from their attachment figure flew in the face of prevailing professional consensus of that era that affectionate interaction was irrelevant or even harmful for normal child development (Watson and Watson, 1928; Blum, 2002). Following separation, both monkeys and children were observed to pass through a period of overt distress or apparent “protest” over the mother’s absence prior to a stage of passive, depressive-like withdrawal termed “despair” or “anaclitic depression” (Spitz and Wolf, 1946; Kaufman and Rosenblum, 1967; Mineka and Suomi, 1978). While initial studies focused on immediate effects, over time it became clear that attachment disruption and other forms of early stress could increase vulnerability for depression, as well as other psychopathologies, in later life (Brown et al., 1977; Agid et al., 1999; Bernet and Stein, 1999). These studies gave rise to “stress diathesis” or “two-hit” models of depression, which propose that the early trauma sensitizes underlying physiological stress-related systems [e.g., increase central corticotropin releasing factor (CRF) release or augment amygdala activation] so that when the individual is exposed to stressors in adolescence or beyond, they elicit enhanced and or unregulated stress responses that can trigger, segue into, or actually constitute the depressive episode (Gold et al., 1988; Schulkin et al., 1994; Heim et al., 2008).

Animal research into the mediators and mechanisms of these effects has been almost exclusively with rodent models, primarily rats and mice. One approach has been to repeatedly deprive rat or mouse pups of maternal stimulation for several hours (Plotsky and Meaney, 1993) rather than the 3–15 min typical of early-handling studies. The longer separations result in an overall reduction in maternal care, rather than the increase seen following handling. A second approach has been to limit the dam’s nesting material, which indirectly disrupts maternal behavior as the female attempts to construct a nest with insufficient material (Walker et al., 2017). For both the several-hour separation and the reduced nest material procedure it is thought that the change in maternal care patterns—either absent during prolonged separations or altered (e.g., fragmented, less predictable, rougher care) when nest material is limited—accounts for most observed effects (Vetulani, 2013; Walker et al., 2017). These studies have been extremely productive and illuminating, showing that disrupted maternal care can produce counterparts to many of the symptoms/responses of human adolescents and adults who have undergone early-life stress (e.g., Walker et al., 2017).

## Attachment and the Guinea Pig

While altered care-giving appears to be a major element of the stress experienced by human children of abusive or neglectful parents, rodent models that manipulate maternal care do not assess another potentially critical aspect of disturbed parent-child interactions, that is, the absence of the attachment figure *per se*. To address this aspect of early-life stress, we have used a guinea pig model. Because of differences in pup development and maternal care, guinea pigs offer a valuable counterpart to studies with rats and mice. In contrast to the extremely altricial state of newborn laboratory rats and mice, guinea pigs are born fully furred with their eyes and ears open. They are capable of independent locomotion soon after birth and can nibble solid food and drink from a water bottle within about a day (Schiml and Hennessy, 1990). Thermoregulatory abilities develop rapidly (Blatteis, 1975; Fewell et al., 1997). Maternal behavior, by contrast, is very passive. The only obvious active maternal care-taking behavior is licking of the pups, and this occurs primarily during the first week of life (König, 1985; Hennessy and Jenkins, 1994). There is no maternal nest and, because mothers do not retrieve pups, it is up to the pup to approach the mother. The pup initiates nursing bouts, with the mother only adjusting body position to provide access to her teat (Hennessy and Jenkins, 1994). It is no surprise then that the young are strongly attracted to the mother from the day of birth. Indeed, pups display evidence of attachment to their mother in terms of recognition, preference, and distressful behavioral and physiological responses to separation (Pettijohn, 1979; Hennessy and Ritchey, 1987; Jäckel and Trillmich, 2003).

**Figure 1:**
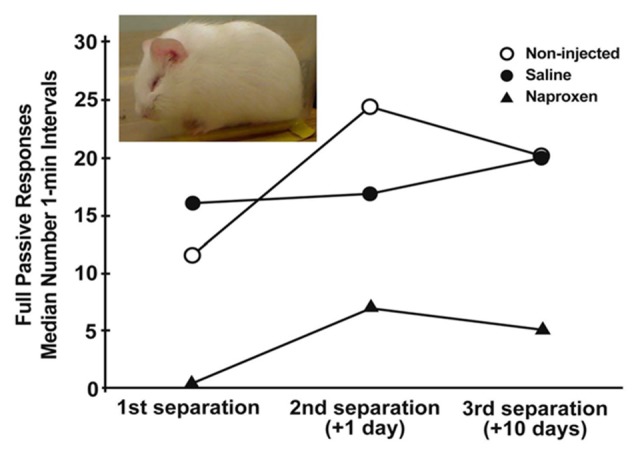
Median number of 1-min intervals guinea pig pups spent exhibiting the depressive-like crouched stance with eye-closure and extensive piloerection (inset) during three, 3-h separations over a 11 days period. Pups were either not injected or were injected for the 3 days prior to the initial separation with either saline vehicle or 14 mg/kg naproxen in saline. Depressive-like behavior increased over separations across all groups (*p* < 0.005). Further, the group given naproxen showed less of the behavior during each separation than did non-injected and saline-injected controls (*p*’s < 0.005; figure derived from Hennessy et al. (2015b) without permission required).

A pup placed alone into a brightly lit novel environment for several hours exhibits HPA and other physiological signs of stress, as well as active “protest,” consisting of high-pitched vocalizations and locomotor activity, followed by a passive second stage characterized by an inactive crouched stance, ptosis or sleepiness, extensive piloerection, and little apparent interest in the pup’s surroundings (Figure 1, insert; Hennessy et al., 1995). The presence of the mother greatly suppresses or eliminates the passive responses, whereas a sibling or unfamiliar adult female or male has less or no effect (Hennessy and Morris, 2005; Hennessy et al., 2015a). Although weaning typically occurs at about 25 days of age, the young will continue to show this two-stage pattern of separation response up until at least early adolescence (Hennessy and Morris, 2005). Because of the physical maturation of the young guinea pig, the several-hour exposure to the novel environment poses no physical threat, and the mother reduces the pup’s responses without exhibiting apparent maternal care. This paradigm, therefore, offers a means to assess the immediate and lasting effects of the psychological variable of presence or absence of the attachment figure.

## Sickness and Separation

On its face, the comparison of the two-stage, active/passive response of guinea pig pups with the “protest” and “despair” of separated primate infants may appear superficial. The primate infants were separated from their attachment figures for a day to weeks, whereas in our work with guinea pigs, the pups were only alone for several hours. For this reason, we initially were hesitant to refer to the response as “depressive-like.” However, the appearance of these animals—inactive, a hunched posture, ptosis or sleepiness, extreme piloerection, and little apparent interest in their surroundings—bore a great similarity to “sickness behavior” as described in others species (Hart, 1988). It was the notion that stress can sometimes induce sickness behavior (Maier and Watkins, 1998) together with growing evidence that sickness behavior is associated with depression (Maes, 1993; Connor and Leonard, 1998) that first led us to focus on the depressive-like quality of the second, passive stage of guinea pig separation, and its possible mediation by inflammatory processes.

Sickness behavior, whether induced by pathogens or by stressors, involves activation of the innate immune system and a systemic inflammatory response with neuroimmune signaling molecules of CNS or peripheral origin acting in critical brain regions to induce behavior change (Maier and Watkins, 1998; Aubert, 1999; DiSabato et al., 2016). Sickness behavior is one component of the broader reaction known as just sickness or, more formally, as “the acute phase response.” Physiological components of sickness include fever, enhanced circulating levels of proinflammatory cytokines and related signaling molecules, production of “acute phase proteins” by the liver, and activation of both sympathetic nervous system and HPA axis. Sickness is adaptive, with fever enhancing the effectiveness of the immune response (Hart, 1988; Maes et al., 2012). Other components of sickness, including sickness behaviors, serve largely to support fever by generating or conserving warmth and conserving energy for the energetically demanding process of fever induction (Hart, 1988). The utility of sickness during stress is less clear, though probably involves preparatory responses in the event the stressor encountered leads to physical injury (Maier and Watkins, 1998) or as a means to promote recovery once the stress challenge has abated (Deak et al., 2017). Sick animals have little interest in otherwise enjoyable activities; they have reduced appetite and exhibit cognitive impairment (Hart, 1988; Maier and Watkins, 1998). In humans, these elements of sickness overlap with symptoms of depression (Maes et al., 2012). Administration of a proinflammatory cytokine as a chemotherapy agent induces sickness (Capuron and Miller, 2004), and depressives often have elevated levels of cytokines in their blood (Young et al., 2014). Although sickness is an adaptive response (Hart, 1988; Maier and Watkins, 1998), it appears that sickness that is intense or does not readily resolve provides a background condition favoring the development of clinical depression (Raedler, 2011; Frank et al., 2016; Reus and Dantzer, 2016).

These considerations prompted us to conduct a series of studies to test whether separated guinea pigs were, in fact, exhibiting stress-induced sickness behaviors. For a start, we needed to show that the behaviors that suggested physical illness to us were, in fact, behaviors guinea pigs show when the innate immune system is activated. We, therefore, injected pups with either saline vehicle or one of two doses of lipopolysaccharide (LPS). LPS is derived from the cell wall of gram negative bacteria so that it potently activates the innate immune system, but without the potential confounds associated with administration of a replicating pathogen. We found LPS induced dose-dependent increases in each of the three measured components of the passive stage (immobile crouched stance, eye-closure, piloerection), indicating that the behavioral response to several hours of separation was the same as occurring during frank sickness (Hennessy et al., 2004). But what about the physiological components of sickness? If separation induces sickness behavior, then we should also expect separation to result in some of the physiological signs of sickness, such as fever. This prediction was confirmed initially in studies with a rectal probe and later with a telemetry system. Our separation procedure induced a small but consistent increase in core body temperature that could not be accounted for by increased physical activity in the test cage (Hennessy et al., 2004, 2010b). Additional work also found the separation procedure to increase gene expression of the proinflammatory cytokine tumor-necrosis factor alpha (TNF-α) in spleen (Hennessy et al., 2007a), another physiological sign of sickness.

Since sickness essentially is a systemic inflammatory response, and we proposed that the depressive-like behaviors guinea pig pups exhibit during separation are sickness behaviors, it should be possible to suppress depressive-like separation responses with anti-inflammatory compounds. This prediction also was confirmed. Pups administered anti-inflammatory agents prior to separation exhibited significantly less, passive, depressive-like behavior. This was true both for broad-based anti-inflammatory compounds [alpha-MSH, interleukin-10 (IL-10)] as well as for more specific cyclooxygenase (COX) inhibitors that target prostaglandin synthesis (indomethacin, naproxen; Schiml-Webb et al., 2006; Hennessy et al., 2007b, 2015b; Perkeybile et al., 2009).

In addition to separation and LPS, another manipulation that we had found to induce the passive, depressive-like response was peripheral injection of CRF (Becker and Hennessy, 1993). In fact, this was the first substance we found to elicit the depressive-like response. Subsequent early studies established that the behavioral response to CRF injection did not appear due to ACTH, cortisol (the primary glucocorticoid of the guinea pig), or β-endorphin released downstream of the CRF (Hennessy et al., 1991), nor could it be accounted for by motoric incapacity or the drop in blood pressure that can follow CRF injection (Becker and Hennessy, 1993; Hennessy et al., 1995). Once we suspected that the depressive-like response was the result of increased inflammatory activity, we asked whether the effect of CRF could be moderated with administration of anti-inflammatory agents, which indeed was found to be the case (Schiml-Webb et al., 2009; Hennessy et al., 2011a). These findings are consistent with studies showing that peripheral CRF has certain pro-inflammatory properties (Webster et al., 1996; Singh et al., 1999), and may reflect a convergent mechanism by which the stress of early separation promotes the expression of depressive-like behavior.

In sum, these studies strongly indicate that the crouched stance, prolonged eye-closure and extensive piloerection elicited by maternal separation reflect sickness responses in young guinea pigs. Intriguingly, early investigators remarked in passing that the behavior of maternally separated monkeys (Kaufman and Rosenblum, 1967), as well as institutionalized children (Spitz and Wolf, 1946), suggested physical illness. Together, it does not seem unlikely that the separation response historically referred to as “despair” or “anaclitic depression” may in large part be comprised of sickness behavior resulting from absence of the attachment figure.

## Inflammatory Processes as Mediators of Early-Experience Effects

We reasoned that if separation from the attachment figure in early life elicits neuro-immune activation leading to increased inflammatory activity that underlies the behavioral reaction of the infant, and then perhaps these same underlying processes also contribute to the long-term effect of early attachment disruption (Hennessy et al., 2010a). In children, this would include increased vulnerability to depression during adolescence and beyond. From a stress-diathesis or “two-hit” perspective, a stress-induced inflammatory reaction might be the, or one of the, stress-responses that becomes sensitized so as to elicit a stronger and/or more-prolonged behavioral response when exposed to subsequent stressors (i.e., second “hits”) at later ages. The first guinea pig finding indicating sensitization was the observation that two, 3-h separations at a 24-h interval increased the number of 1-min intervals that guinea pig pups spent exhibiting the passive, depressive-like response on the second day of separation (Hennessy et al., 2010b). In the same study, we observed that fever also sensitized, rising more rapidly and showing a more robust elevation over control values the second day. Although supporting our hypothesis in principle, sensitization over the course of a single day is not a compelling model for the sensitization that is hypothesized to occur between the time of trauma in childhood and the onset of depression in adolescence or later. In subsequent published studies we, therefore, added a third, 3-h separation up to 10 days after the first (i.e., ~day 31) and observed the same pattern of increased depressive-like behavior and fever, effects that could not be accounted for by the older age of animals at the time of the third separation (Schneider et al., 2012; Yusko et al., 2012). While we have not yet maintained previously separated and non-separated guinea pigs for testing in full adulthood, it is clear that sensitization of passive, depressive-like behavior persists past weaning and into the juvenile or early adolescent phase of life, lasting well beyond what would normally be expected from a relatively brief stress challenge.

The sensitization of fever provides indirect evidence for the hypothesis that the sensitization process involves an enhancement of inflammatory activity. To test this idea more directly, we administered either the anti-inflammatory cytokine IL-10 or artificial cerebrospinal fluid vehicle to pups through a surgically implanted intracerebroventricular cannula prior to a 3-h separation. Pups were then separated a second time 24 h later. Whereas the vehicle controls showed an increase in depressive-like behavior from the first separation to the second, IL-10 administration blocked the sensitization response (Hennessy et al., 2011b). A second set of experiments used a less-invasive procedure of peripheral injection of the COX-inhibitor naproxen, examined sensitization over 10 days, and assessed the fever response as well as behavior. As seen in Figure 1, pups that were injected with a 14 mg/kg dose of naproxen for three consecutive days prior to the initial separation exhibited significantly less depressive-like passive behavior than did saline-injected and non-injected controls across all 3 days of testing (Hennessy et al., 2015b). Fever also was blunted with naproxen, though only with a 28 mg/kg dose. Together, these two sets of findings provide strong evidence for an involvement of inflammatory processes in the sensitization of depressive-like behavior over repeated separation.

One potential problem with a model such as described thus far is the issue of “trans-situationality” (Maier and Watkins, 2005). That is, our model is intended to shed light on how attachment disruption in early life increases vulnerability for major depressive disorder. Depression can manifest in a variety of contexts and situations, but our data presented to this point is limited to the guinea pig’s experience during isolation in a test cage affecting its later behavior in the same situation—isolation in a test cage. Therefore, to examine whether the sensitization could be observed in a different situation, we turned to the Forced Swim Test. This test is the most widely used preclinical screen for antidepressants. Time spent immobile in the forced swim has high predictive validity in that immobility is selectively reduced by a range of antidepressant medications, but not other drugs (Cryan et al., 2005; Czéh et al., 2016). Although this test has been employed almost exclusively with rats and mice, it has been validated in tests with guinea pigs in which selective reduction of immobility with antidepressant compounds was documented (Wicke et al., 2007; Rex et al., 2008). Thus, to determine if the effect of previous separation would generalize to another depression-related measure, we either separated or did not separate preweaning guinea pig pups for 3 h in the standard way and, beginning 24 h later, tested them for 5 min in the Forced Swim Test on three consecutive days. We found that the previously separated pups showed more immobility across the 3 days of testing than did pups that had not previously been separated, indicating that the effects of prior separation were not simply task-specific, but rather exhibited trans-situational generality (Hennessy et al., 2017b). Together with previous findings, the results in the Forced-Swim Test suggest early separation did indeed affect underlying neural processes common to human depression.

## But What Is the Sensitization Process?

At about the same time as the experiments described here were conducted, evidence was accruing from human studies linking attachment disruption and other forms of early-life stress to the development of inflammatory-mediated depression (Slavich and Irwin, 2014). For instance, early stress was found to be related to measures of systemic inflammation (e.g., Bertone-Johnson et al., 2012; Gouin et al., 2012; Slopen et al., 2013), enhancement or sensitization of inflammation (Miller and Chen, 2010), and the occurrence of depressive illness (Danese et al., 2008; Miller and Cole, 2012) in adolescence and beyond. It appears likely then that inflammatory factors underlie increased vulnerability for depression in humans as well as increased depressive-like behavior in guinea pigs. However, the actual process or processes involved, and whether they vary across species, remains unclear.

Although glucocorticoids have powerful anti-inflammatory effects, recent studies in adult rats indicate that increased inflammatory activity can be triggered by prior elevation of glucocorticoid levels. Adult rats exposed to a regime of tail shock showed increased release of proinflammatory signaling molecules when injected with LPS the next day—that is, a potentiated or sensitized response. Interestingly, a comparable increase in the signaling molecule response to LPS was observed if the rats were simply injected the first day with a dose of corticosterone (the primary glucocorticoid in the rat) that mimicked the increase in circulating corticosterone levels produced by the shock (Frank et al., 2016). Thus, corticosterone elicited the same potentiated response as did prior stress, suggesting that HPA axis responses may prime inflammatory responses to subsequent challenges.

In light of these findings, we recently examined the effect of cortisol injection on the passive, depressive-like response of guinea pig pups. Pups reliably exhibit a plasma cortisol elevation upon isolation in a novel cage (e.g., Hennessy and Moorman, 1989). Since an initial separation enhances the passive behavioral response and fever following a second separation 24 h later (Hennessy et al., 2010b), we asked whether a cortisol injection could mimic the effect of the first separation on behavior and fever the next day. Three groups of 16 pups each (eight males, eight females) were implanted with telemetry devices to measure core temperature. At least 4 days later (20–24 days; Mean age = 23.0–23.9/group), pups were injected subcutaneously with saline, or 2.5 or 10.0 mg/kg cortisol. We returned the pups to their cages, and 24 h later separated them in a novel environment, measuring core temperature throughout the test and recording the number of minutes in which the pups displayed the passive, depressive-like behavior during 0–30, 60–90, and 150–180 min. One to 3 days following the test, a subset of animals was used to examine the effect of the cortisol injections or just separation on circulating cortisol levels. Blood was collected non-invasively from ear veins, prior to the separation, as well as 1 and 3 h later, for cortisol radioimmunoassay. These experimental procedures, presented here for the first time, were in compliance with Public Health Service guidelines and were approved by the Wright State University Institutional Animal Care and Use Committee.

**Figure 2:**
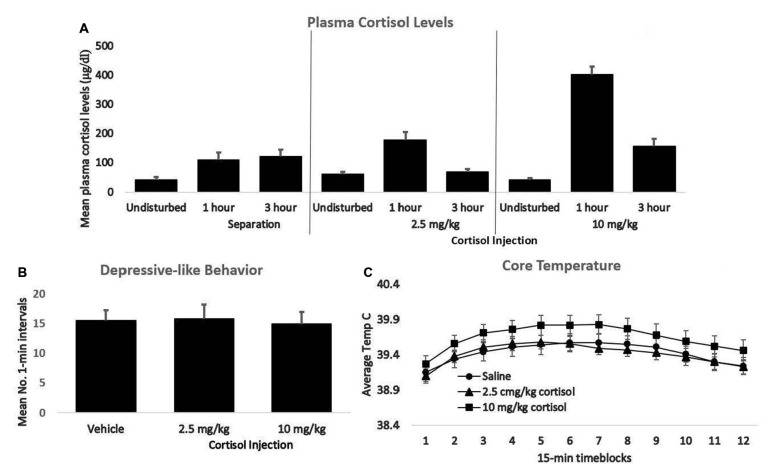
**(A)** Mean plasma cortisol levels of young guinea pigs 1 and 3 h following either separation or injection of either 2.5 or 10 mg/kg of cortisol. For each group, cortisol values following no prior disturbance are presented for comparison. **(B)** The mean number of 1-min intervals pups that had been either undisturbed or injected with 2.5 or 10 mg/kg cortisol spent exhibiting the depressive-like crouched stance with eye-closure and extensive piloerection during separation the following day. **(C)** Mean core body temperature of the same three groups depicted in panel B during 15-min time blocks of the 3-h separation. In all panels, vertical lines represent standard errors of the means. There was no significant difference across groups for either behavior or core temperature.

The 10.0 mg/kg dose of cortisol produced plasma elevations that were clearly in the pharmacological range, whereas the 2.5 mg/kg dose resulted in cortisol elevations that roughly approximated those seen during separation (Figure 2A). Nonetheless, neither the low- nor the high-dose injection produced any greater passive, depressive-like behavior or elevation of core temperature in males or females than did saline injection (Figures 2B,C). Thus, for guinea pig pups an elevation of glucocorticoids was not sufficient to trigger the sensitization process. These results suggest a difference in the basic mechanism underlying sensitization in adult rats subjected to shock vs. infant guinea pigs isolated in novel surroundings. Norepinephrine is a second stress mediator that has been implicated to varying extents in the sensitization of inflammatory processes in adult rats and mice (Blandino et al., 2006; Reader et al., 2015). The role of norepinephrine, either alone or in conjunction with cortisol, in triggering sensitization of inflammatory responses in guinea pig pups would be worthy of further study.

Another recent study of the sensitization process (Hennessy et al., 2019) examined changes in expression of central signaling molecules following early separation. We tested the hypothesis that sensitization of behavior and fever induced by prior separation would involve increased gene expression of neuroimmune mediators in a critical brain region—the hypothalamus. Approximately 3-week-old pups were either separated or not on two consecutive days, and then 10 days after the first separation, they were injected with either a modest dose of LPS (25 μg/kg) or saline, and then isolated in a novel cage prior to harvesting tissue at 30, 60, or 120 min. LPS greatly enhanced expression of the chemokines MCP-1 and CXCL1, as well as the prostaglandin synthesizing enzymes mPGES and COX-2 at 120 min. These enzymes were of particular interest since COX inhibitors can reduce depressive-like behavior during separation (Hennessy et al., 2007b, 2015b). However, for each of these four molecules, prior separation blunted, rather than enhanced, the effect of LPS. While unexpected, these results are not inconsistent with past findings. Recall that blocking inflammation prior to a first separation moderates or eliminates sensitization of depressive-like behavior (e.g., Figure 1). These data suggest that releases of inflammatory-related signaling molecules are involved in the *initiation* of the sensitization process. This initial response then appears to produce a different class of secondary effects, either inflammatory (e.g., increased sensitivity of receptors or downstream signaling cascades) or non-inflammatory (e.g., altered limbic activity) that then mediates the actual sensitized response. In either case, the reduced expression of neuroimmune signaling molecules in previously separated animals following LPS injection may represent a compensatory effect preventing even greater sensitization.

In all, our investigation of the actual process of sensitization in guinea pig pups separated from the attachment figure suggests some fundamental differences compared to what is known about sensitization of inflammatory processes following electric shock in adult rats. In infant guinea pigs, it appears that glucocorticoids play a lesser role in initiation of the sensitization process, and increased expression of neuroimmune signaling molecules seems unlikely to be the proximal cause of the sensitized behavioral response. Whether these apparent distinctions are due to differences in species, developmental stage, stressor, test procedures, or some combination remains to be tested.

## Return to Primates

The choice of the guinea pig model was originally inspired by primate research demonstrating the impact of the attachment figure on biobehavioral processes and their development. The similarities in attachment and separation responses of guinea pigs and monkeys (Hennessy, 2003) suggested that guinea pig research might serve as a complement to primate studies, not only by providing a broader comparative basis, but also by addressing questions more rapidly and economically than would be possible with nonhuman primates, and with adequate sample sizes not always available for nonhuman primate studies. In this way, hypotheses might be generated that could then be tested in nonhuman primates. Yet, it was unclear how many of the results reported here could be tested in monkeys without return to the severe procedures with separated infants of decades ago. However, a possible means of examining some of our hypotheses in principle, but with adult monkeys, arose in collaboration with investigators at the California National Primate Research Center. Adult rhesus macaques brought temporarily from large outdoor social groups to individual or paired indoor housing for experiments, treatment, or other reasons were noted to occasionally display a hunched posture with apparent disinterest in the environment that was reminiscent of the posture of the separated monkey infants from years past. This observation was surprising in that such a response is not considered typical of healthy adult rhesus under normal conditions. We then re-examined data from an unrelated project in which adult male monkeys brought from outdoor field cages to indoor, individual housing were observed by videotape with no human in the room. In just 10 min of observation during the first week, 18 of 26 monkeys were seen in the hunched posture (Hennessy et al., 2014). We speculated that this response may be more common during moderately stressful conditions than generally believed because observations typically are made by a live observer, and it is well-established that rhesus respond defensively to the approach of a human (e.g., Hamel et al., 2017).

**Figure 3:**
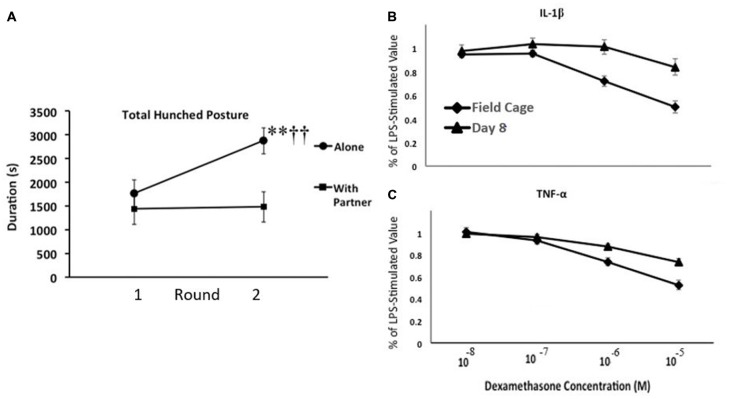
**(A)** Mean number of seconds that adult male rhesus brought from spacious outdoor social groups to indoor housing either alone or with a partner exhibited the depressive-like hunched stance during 2, 8-day rounds of indoor housing at a 2-week interval. Monkeys housed alone exhibited a sensitization of depressive-like behavior from the first to the second round of indoor housing (**p < 0.001), so that they exhibited more of the behavior than did pair-housed monkeys during the second round (^†^^†^*p* < 0.001), but not the first. **(B,C)** Mean percentage of lipopolysaccharide (LPS)-stimulated values of interleukin-1β (IL-1β; **B**) and tumor-necrosis factor alpha (TNF-α; **C**) following addition of various doses of dexamethasone. Blood was collected when monkeys were housed in field cages in large social groups and following 8 days of indoor housing. The higher doses of dexamethasone had less of a suppressive effect on both cytokines following 8 days indoors (*p*’s < 0.01). For both panels, vertical lines represent standard errors of the means (figure derived from Hennessy et al., 2017a with permission).

We, therefore, conducted an experiment with adult male monkeys to confirm these observations and to test some of the general notions derived from our guinea pig studies (Hennessy et al., 2017a). Specifically, we asked whether moving adult rhesus from spacious, outdoor, mixed age/sex social groups to indoor, individual- or paired-housing would reliably elicit the hunched “depressive-like” response; if the response of the monkeys, like that of the separated guinea pig pups, would sensitize with repeated indoor housing apart from the social group; and if the behavioral response was associated with increased inflammatory activity. Monkeys were brought indoors either alone or with a younger affiliative partner (younger sibling or juvenile male) for 8 days on two occasions at a 2-week interval. Behavior was observed throughout the indoor stay via video with no human in the room, and blood samples were collected during, as well as well as prior to, each round of indoor housing to assess inflammatory activity and cortisol. During the first round indoors, both isolated and paired animals spent about a third of observations in the hunched posture (Figure 3A). The response of the pair-housed animals was unchanged during the second separation, whereas the response of the individually housed animals sensitized. These monkeys spent well over half of the observation time in the hunched posture. This behavioral response was accompanied by inflammatory changes. Of particular interest, we found a change in the ability of glucocorticoids to suppress proinflammatory cytokine levels. Blood collected from monkeys prior to indoor housing and at the end of each round was stimulated with LPS, and then different concentrations of the synthetic glucocorticoid dexamethasone were added to different aliquots of the LPS-stimulated blood prior to analysis for cytokine levels. Regardless of whether monkeys had been isolated or pair-housed indoors, 8 days of indoor housing apart from the social group reduced the ability of glucocorticoids to suppress the LPS-induced increase of the two proinflammatory cytokines measured—IL-1β and TNF-α (Figures 3B,C). Plasma cortisol levels, in contrast, were comparable during indoor housing for the two groups of monkeys. Reduced resistance to glucocorticoids is one of the inflammatory measures that has been observed in children who were raised under harsh conditions (Miller and Chen, 2010). We also obtained evidence for a linkage between the behavioral and cytokine responses. For the isolated, but not pair-housed, animals, duration of the hunched posture was positively and significantly correlated with each of the three cytokines measured (IL-1β, TNF-α, and the anti-inflammatory cytokine IL-10) during each round of indoor housing apart from the social group (Pearson correlation coefficients from +0.628 to +0.768).

This monkey experiment and the guinea pig studies differed in many obvious ways in terms of procedures, measures, and the developmental stage at which animals were tested. Nonetheless, the monkey results demonstrate that, as in guinea pig pups, a period of social separation in novel surroundings can elicit a “depressive-like” hunched posture in adult male rhesus. The behavioral response is associated with increased inflammatory activity and seems likely to represent a stress-induced sickness response. Further, for the individually housed monkeys, as for isolated guinea pig pups, the behavioral response sensitized with repeated testing. These results provide some confidence that at least the broad outlines of the phenomenon we have been examining in guinea pigs are translatable to primate species. The findings also suggest that what amounts to a routine animal husbandry procedure might serve as a nonhuman primate model to assess means by which social isolation can promote vulnerability to depression. It bears repeating that sickness behavior, including stress-induced sickness behavior, is considered an adaptive response, but one that can progress to depressive illness. For that reason, what seems best modeled by either the guinea pig or monkey studies is increased vulnerability for depressive illness, or a depression-prone phenotype, rather than depression itself.

Certainly, a primary goal of future research must be a better understanding of central mechanisms underlying the sensitization process. This work will need to address the relative contribution and potential interaction of the various brain regions strongly implicated in stress and depressive illness (e.g., prefrontal cortex, amygdala, hippocampus, hypothalamus). Further, our studies to date have not emphasized questions of male-female differences. The monkey study used only males, and while both males and females typically have been included in our guinea pig experiments, these experiments rarely have been sufficiently powered to detect any but large magnitude differences between the sexes. It is not surprising then that, with the exception of seemingly greater sensitivity of females to LPS (Hennessy et al., 2013), we have no evidence for male-female differences in the guinea pig studies. However, given the higher incidence of depression in women than in men, a better understanding of sex differences in our primate and guinea pig models is another major goal of future work.

## Final Considerations

Attempting to use a guinea pig or a rat to study a phenomenon as complex as the means by which poor childrearing and disrupted attachment processes can lead to susceptibility to major depressive disorder in adulthood can appear quixotic. Depression, with its intertwined emotional and cognitive components, is likely a uniquely human condition. Perhaps macaques can experience something akin to human depression, but rodents almost certainly cannot. Similarly, the relationship between mother and child would seem to have only very limited overlap with the caretaking by a lactating rat. Yet, early attachment disruption or altered maternal care leading to vulnerability for depressive illness in adolescence or adulthood is an example of developmental plasticity, a trait that is common across vertebrate species (Snell-Wood, 2013; Bateson et al., 2014).

It is widely accepted that if cues in the early environment afford information that somehow presages an otherwise unpredictable adult environment, altering the trajectory of development to better match the likely adult environment will have fitness value, and so will be subject to natural selection (Fischer et al., 2014; Fawcett and Frankenhuis, 2015). Interestingly, these “cues” afforded by the early environment often involve stressful events. For instance, in red squirrels, detecting auditory signals of nearby conspecifics (indicating heightened population density) leads to accelerated early growth of newborns—a trait that is only adaptive under high density conditions, and which appears to be mediated by an increase in glucocorticoid secretion (Dantzer et al., 2013). However, plasticity has biological costs and may involve pleiotropic effects, not all of which necessarily are adaptive, and the enhanced fitness stemming from an altered developmental trajectory results only if the predicted adult environment is, in fact, encountered in adulthood. Increased vulnerability for depression following early-life stress may then stem from a cost or pleiotropic effect of plasticity induced by early stress, or to a mismatch between “predicted” and encountered adult environment (Schmidt, 2011; Nederhof et al., 2013).

Viewed from this perspective, the value of animal models of early-life stress is not so much in how closely they can mimic human depression, or which model is “best” in this regard, but rather in what they can tell us about how early stress alters key developmental processes that are conserved across mammalian species, and which in humans, increase vulnerability for psychopathology. Given the potential adaptive benefit of altering one’s developmental trajectory to match the future environment, one would expect multiple, and possibly redundant, mechanisms of plasticity to evolve in response to a variety of early “cues” (i.e., stressful events). For this reason, multiple animal models that can both maximize focus on the effects of particular early cues (e.g., altered early care vs. absence of attachment object) and provide evidence of conservation across species, should be valuable in unraveling the basic processes underlying maladaptive effects of early experience in humans.

## Author Contributions

MH wrote the initial draft. PS and TD edited the draft. KB and NB conducted the previously unpublished study.

## Conflict of Interest Statement

The authors declare that the research was conducted in the absence of any commercial or financial relationships that could be construed as a potential conflict of interest.
